# Crystal Structure of the Complex mAb 17.2 and the C-Terminal Region of *Trypanosoma cruzi* P2β Protein: Implications in Cross-Reactivity

**DOI:** 10.1371/journal.pntd.0001375

**Published:** 2011-11-01

**Authors:** Juan Carlos Pizarro, Ginette Boulot, Graham A. Bentley, Karina A. Gómez, Johan Hoebeke, Mireille Hontebeyrie, Mariano J. Levin, Cristian R. Smulski

**Affiliations:** 1 Unité d'Immunologie Structurale, Institut Pasteur, Paris, France; 2 Centre National de la Recherche Scientifique, Unité de Recherche Associée 2185, Paris, France; 3 Laboratorio de Biología Molecular de la Enfermedad de Chagas, INGEBI-CONICET, Buenos Aires, Argentina; 4 UPR9021 du CNRS, Strasbourg, France; 5 Institut Pasteur, Paris, France; National Institutes of Health, United States of America

## Abstract

Patients with Chronic Chagas' Heart Disease possess high levels of antibodies against the carboxyl-terminal end of the ribosomal P2ß protein of *Trypanosoma cruzi* (TcP2ß). These antibodies, as well as the murine monoclonal antibody (mAb) 17.2, recognize the last 13 amino acids of TcP2ß (called the R13 epitope: EEEDDDMGFGLFD) and are able to cross-react with, and stimulate, the ß1 adrenergic receptor (ß1-AR). Indeed, the mAb 17.2 was able to specifically detect human β1-AR, stably transfected into HEK cells, by flow cytometry and to induce repolarisation abnormalities and first degree atrioventricular conduction block after passive transfer to naïve mice. To study the structural basis of this cross-reactivity, we determined the crystal structure of the Fab region of the mAb 17.2 alone at 2.31 Å resolution and in complex with the R13 peptide at 1.89 Å resolution. We identified as key contact residues on R13 peptide Glu3, Asp6 and Phe9 as was previously shown by alanine scanning. Additionally, we generated a model of human β1-AR to elucidate the interaction with anti-R13 antibodies. These data provide an understanding of the molecular basis of cross-reactive antibodies induced by chronic infection with *Trypanosoma cruzi*.

## Author Summary

*Trypanosoma cruzi* is a protozoan parasite responsible for Chagas disease. Chronic Chagas' heart disease (cChHD) is not only the most frequent and severe consequence of the chronic infection by *T. cruzi*, but is also the main cause of cardiomyopathy in South and Central America. Patients with cChHD possess high levels of antibodies against the carboxyl-terminal tail of the ribosomal P proteins of *T. cruzi* (called the R13 epitope). These antibodies, as well as the murine monoclonal antibody (mAb) 17.2, are able to cross-react with, and stimulate, the ß1 adrenergic receptor (ß1-AR). Indeed, the mAb 17.2 was able to specifically detect human β1-AR and induce some of the classical cardiac symptoms after passive transfer to mice. To study the structural basis of this cross-reactivity, we determined the crystal structure of the Fab region of the mAb 17.2 alone and in complex with R13. Additionally, we generated a model of human β1-AR to elucidate the interaction with anti-R13 antibodies in order to understand the molecular basis of cross-reactive antibodies induced by chronic infection with *T. cruzi*.

## Introduction

*Trypanosoma cruzi*, the etiological agent of Chagas' disease, affects approximately 10–12 million people from different endemic regions in Latin America [1], killing more than 15,000 each year [2], [3]. It is also carried by hundreds of thousands of people in Europe (especially Spain and Portugal), the United States, Canada, Japan and Australia, mostly by Latin American immigrants. Accordingly, infection control of blood banks has been recently implemented outside endemic regions [4]. Chagas' disease shows a variable clinical course, which ranges from an acute phase, with parasitemia and asymptomatic features, to serious chronic symptomatic stages characterized by low or no parasitemia, positive serology and involving clinical cardiac, gastrointestinal or neurological disorders [3], [5]. The cardiac form, referred as chronic Chagas' heart disease (cChHD), is not only the most frequent and severe consequence of the chronic infection by *T. cruzi*, but is also the main cause of cardiomyopathy in South and Central America [6]. Among other clinical features, cChHD is an arrhythmogenic cardiomyopathy with high prevalence of right bundle branch block, left anterior hemi block, sinus node dysfunction and complex supraventricular arrhythmias [7], [8]; mega disorders of colon or esophagus and neurological disorders are less frequent (around 5% of the infected people) [9], [10]. To date, the mechanisms of the pathophysiology of Chagas' disease are not completely understood and two main hypotheses have been proposed: the first is based on the essential role of the parasite in tissular damage while the second argues for an auto reactive process resulting from an impaired immune response associated with molecular mimicry [11], [12], [13]. Different experimental approaches have shown that both mechanisms may be involved in the chagasic pathology because of the persistence of the parasite in chronic phase [14] and of the presence of parasite antigens carrying epitopes common to host molecules [15].

In 1989, Levin et al. selected and cloned the first parasite molecule, called JL5, presenting a molecular mimicry of a human molecule, using serum from a chronically infected person with cChHD [16], [17]. The fine epitope recognized by patients with cChHD was located at the C-terminal end of the *T. cruzi* ribosomal P2β protein (TcP2β) and was named R13 (EEEDDDMGFGLFD) [15], [18], [19], [20]. This highly antigenic acidic epitope bears similarity to an acidic motif (AESDEA) on the second extracellular loop of cardiac β1-adrenergic receptor (β1-AR) [21], [22]. In addition, a significant correlation between the high level of anti-R13 antibodies (Abs) and ventricular arrhythmias was observed [23], consistent with the hypothesis that R13-specific anti-TcP2β Abs are able to cross-react with and stimulate the β1-AR [19], [20], [21], [22], [23], [24], [25]. Alanine-mutation scanning experiments on the R13 epitope using different immuno-purified anti-R13 Abs illustrated the complexity of the anti-R13 humoral response since each of the eight anti-R13 Ab preparations presented a unique epitope recognition pattern [23]. Despite this extreme heterogeneity, it was possible to determine a common reactivity profile where Glu3, and to a lesser extent, Asp6 and Phe9 were essential [23]. Indeed, the C-terminal end of the human ribosomal P proteins has one single amino acid change in the third residue (Glu3Ser), a change that diminished the affinity of mAb 17.2 for the corresponding mammalian peptide by about two orders of magnitude [22].

Mice immunized with different recombinant TcP2β proteins (GST or His fusion proteins) and different adjuvants (CFA or ALU) induced a diverse response along the protein sequence. Strikingly, Abs from infected animals recognized only the C-terminal region of the protein (R13 epitope). These different antiserum showed that only Abs specific for the C-terminus were able to increase the beating frequency of cardiomyocytes from neonatal rats *in vitro* by selective stimulation of the β1-AR [24]. These immunization data led to protocols for the production of a monoclonal antibody directed against the R13 epitope, the mAb 17.2. This mAb was demonstrated to i) recognize a linear epitope of the C-terminal end of TcP2β protein (R13), ii) react with peptides derived from the second extracellular loop of the human β1-AR, iii) induce a dose-dependent increase on the beating frequency of cardiomyocytes in culture that is abolished by bisoprolol, a specific β1-AR antagonist [22], and iv) provoke apoptosis in murine cardiac cell lines, HL-1 [26].

In the present work, we report the three-dimensional structure of the Fab fragment of mAb 17.2 determined by X-ray crystallography, alone and in complex with its cognate peptide epitope, providing a description of structural changes that occur upon binding the antigen. The mAb 17.2 was shown by flow cytometry to specifically detect HEK cells transfected with the human β1-AR. In addition, passive transfer to naïve mice induced some of the classical symptoms of the Chagasic cardiomyopathy, such as repolarisation abnormalities and first degree atrioventricular (AV) conduction block. Finally, we discuss the relationship between epitope mimicry and bystander activity of anti-R13 Abs on the β1-AR using our crystal structure of the Fab 17.2 in complex with a model of the human β1-AR constructed from the turkey β1-AR structure that was recently determined [27].

## Materials and Methods

### 1. Ethics statement

The research was conducted in accordance with the European Community guidelines for use of experimental animals. The IBMC animal house facilities are approved by French veterinary service (#E67-482-2). No surgery has been done on animals. Mice were euthanized according the European Community guidelines.

### 2. Preparation and purification of mAb 17.2

The mAb 17.2 (isotype IgG1, κ) was obtained by immunizing BALB/c mice with recombinant TcP2β [22]. The mAb was purified from ascitic fluid by precipitation with 40% ammonium sulphate at pH 7.4, followed by ion-exchange chromatography on a DEAE-Sephacel (Pharmacia, Sweden) column equilibrated in 17.5 mM NaCl at pH 8.0. The elution was done with 40 mM NaCl at pH 8.0. The sample was then dialyzed against 0.1 M potassium phosphate at pH 7.2.

After addition of 5 mM β-mercaptoethanol and 2.5 mM EDTA, the IgG was treated with papain at an enzyme∶substrate ratio of 1∶100 at 37°C for 2 h. The reaction was stopped by addition of iodacetamide (1 mg/ml) before dialysis against 10 mM sodium phosphate at pH 8.0. The Fab was then added to a DEAE-Sephacel (Pharmacia, Sweden) column equilibrated with the same buffer. Fab 17.2 was eluted with phosphate buffer and then concentrated on a Centricon 10.

### 3. Synthetic peptide

R13 peptide (EEEDDDMGFGLFD, representing the C-terminal region of TcP2β) was synthesized by the solid-phase method of Merrifield [28], with a semi-automatic multi-synthesizer NPS 4000 (Neosystem, France).

### 4. Crystallization of the complex Fab 17.2

Crystals of apo Fab 17.2 and its complex with R13 were grown by vapour diffusion using the hanging drop technique. One volume of Fab 17.2 at 10 mg/ml was mixed with one volume of a solution consisting of 18% (w/v) PEG 8000, 0.1 M sodium cacodylate at pH 6.7, 0.2 M calcium acetate, 12.5 mM Tris-HCl pH 7.5 and 25 mM NaCl. The drop, with a final protein concentration of 4.2 mg/ml, was sealed over a reservoir containing 1 ml of mother liquor. Crystallization of the Fab 17.2/R13 peptide was performed under the same conditions as above with Fab and peptide concentrations of 15 mg/ml and 10 mg/ml, respectively, giving an Ab/antigen molar ratio ∼1/20.

### 5. Crystallographic data and structure solution

Diffraction data were collected on the beam lines ID14-1 (Fab 17.2/R13 complex) and BM30A (apo Fab 17.2) at the European Synchrotron Radiation Facility, Grenoble, France. The two crystal forms belong to space group P2_1_ with very similar unit cell parameters. Diffraction data were integrated and scaled using the programs HKL and SCALEPACK [29]. The structure was solved by molecular replacement with the program AMoRe [30], using antibody variable and constant dimers derived from PDB entry 2igf as search models. Two independent Fab molecules, expected from the unit cell volume, were readily located and the structure was refined using the program autoBUSTER (Global Phasing Ltd.). Manual adjustments between refinement runs and building of the R13 peptide in the complex were performed with the program COOT [31]. Crystallographic parameters, diffraction data statistics and structure refinement results are summarized in Table 1.

**Table 1:** Crystallographic data and refinement statistics.

		Fab 17.2 - R13(EEEDDDMGFGLFD)		Fab 17.2 apo	
Space group		P2_1_		P2_1_	
Cell parameters	abc(Å)	83.0068.2192.11		82.0765.6891.37	
	β (°)	98.4		98.2	
Resolution (Å)		30.0–1.89	1.94–1.89	30.0–2.29	2.34–2.29
Total reflections		282283		144,460	2210
Unique reflections		73,918	3090	41,572	1458
R_merge_		0.077	0.405	0.049	0.170
Completeness (%)		92.0	57.9	94.6	50.7
*δ*		14.6	2.7	22.9	4.2
Redundancy		3.82	3.82	3.47	3.47
B Wilson (Å^2^)		28.8		28.8	
Refinement					
Resolution (Å)		20.0–1.89	1.94–1.89	20.0–2.31	2.37–2.31
R_factor_		0.1795	0.2107	0.1724	0.2013
R_free_		0.2178	0.2459	0.2355	0.3122
rms bonds (Å)		0.010		0.010	
rms angle (°)		1.15		1.25	

### 6. β1-AR competition binding experiments

HEK cells and HEK cells transfected with β1-AR (HEK-β1) were cultured in DMEM (GIBCO, Invitrogen, USA) with 10% FCS, 100 U/ml penicillin, 100 mg/ml streptomycin and 2 mM L-glutamine, using 200 µg/ml hygromycin B (Sigma, USA) for transfected cell maintenance [25].

HEK and HEK-β1 cells were seeded in FACS tubes at 8×10^5^ cell/ml and incubated with mAb 40.14 or 17.2 (500 nM) or with mAb 17.2 (500 nM) pre-incubated for 2 h at 37°C with 10 µM R13 peptide in supplemented DMEM. After 1 h at room temperature, cells were stained with Cy3-conjugated goat anti-mouse IgG (Jackson ImmunoResearch, USA) and propidium iodide (PI) to exclude the dead cell population. Cells were detected using a BD FACSARIA flow cytometer (BD Biosciences) and results were analysed with WinMdi 2.9 software (Copyright 1993–2000 Joseph Trotter). Values are expressed as means ± S.D. (n = 3) and statistical comparisons were performed using One-way and Two-way ANOVA with Bonferroni's Multiple Comparison post test using GraphPad Prism version 5.00 for Windows, GraphPad Software, San Diego California USA, www.graphpad.com. *P*<0.05 was considered statistically significant.

### 7. Electrocardiogram recordings

Three female BALB/c mice, 20 g each, were anesthetized using intraperitoneal Tribromoethanol 150 mg/Kg. The mAb 17.2 was injected intravenously at 200 nM in 200 µl of saline solution (0.9% NaCl). Electrocardiogram (ECG) recordings were performed during 30 min after injection. ECGs were obtained with the six standard leads (I, II, III, AVR, AVL, AVF) at 50 mm/s of paper speed and at 20 mm/mV amplitude using a Fukuda-Denshi Fx-2111 electrocardiograph (Tokyo, Japan) [32]. Electrocardiographic analysis included measurements of heart rate, P wave duration and amplitude, QRS complex duration and amplitude, P-R interval duration and a search for disturbances of rhythm, conduction, and repolarisation.

### 8. Molecular modelling

All procedures were performed with Discovery Studio 2.5 software from Accelrys (San Diego, CA, USA). The crystal structure of the turkey β1-AR (PDB 2VT4) was used as template to build a model of the human β1-AR. We mutated all residues to the human sequence and optimized the conformation of both the mutated residues and any surrounding residues that lay within a cut-off radius of 2 Å. Five models thus obtained were scored by the Discrete Optimized Protein Energy (DOPE). We continued analysis using the lowest energy model (DOPE = −39358.55 kcal/mol), which was superimposed onto a membrane model. The R13 epitope structure was aligned with the second extracellular loop of the humanized β1-AR model to allow positioning of the Ab on the putative cross-reactive epitope. Once the Ab was positioned, a fixed atom constraint was applied to the whole β1-AR with the exception of the extracellular regions and a harmonic restraint was applied to the frame work regions of the Ab. Finally, the complex was subjected to an initial minimization step (max 500), RMS gradient 0.1 Kcal/mol by conjugated gradient; followed by a second minimization step (max 500), RMS gradient 0.0001 Kcal/mol by conjugated gradient. The structure was then submitted to a dynamic simulation including heating (2000 steps), equilibration (1000 steps) and production (1000 steps) at 300 °K with a time step of 0.001 ps under a distance-dependent, dielectric constant, implicit solvent model. A negative intermolecular energy of −430 kcal/mol was obtained for the complex under these conditions. All images were generated using PyMOL Molecular Graphics System, Version 1.0, Schrödinger, LLC.

## Results

### Structure of apo Fab 17.2 and its complex with R13

We determined the crystal structure of the Fab region of the mouse monoclonal antibody 17.2, in the apo form and in complex with the peptide R13 (EEEDDDMGFGLFD), corresponding to the C-terminal epitope of TcP2β. The asymmetric unit of both crystal structures contains two independent apo Fab molecules or Fab-R13 complexes (referred as molecule 1 and 2). The N- and C-termini of R13 show considerable mobility and only the first eleven amino acid residues (EEEDDDMGFGL) in both complexes could be traced in the electron density maps. The two independent Fab molecules of each crystal form show small differences in quaternary structure, as revealed by the difference in elbow angle (angle subtended by the pseudo two-fold axes of the variable and constant dimers). The Fab 17.2 elbow angles are 152/144° and 150/146° for the apo and R13 complex, respectively (molecule1/molecule2), and lie within the range of previously reported values [33]. These reflect the different lattice environments of the two independent molecules in the asymmetric unit since the respective elbow angles for molecules 1 and 2 are common to both crystal forms, which are closely isomorphous.

**Figure 1:**
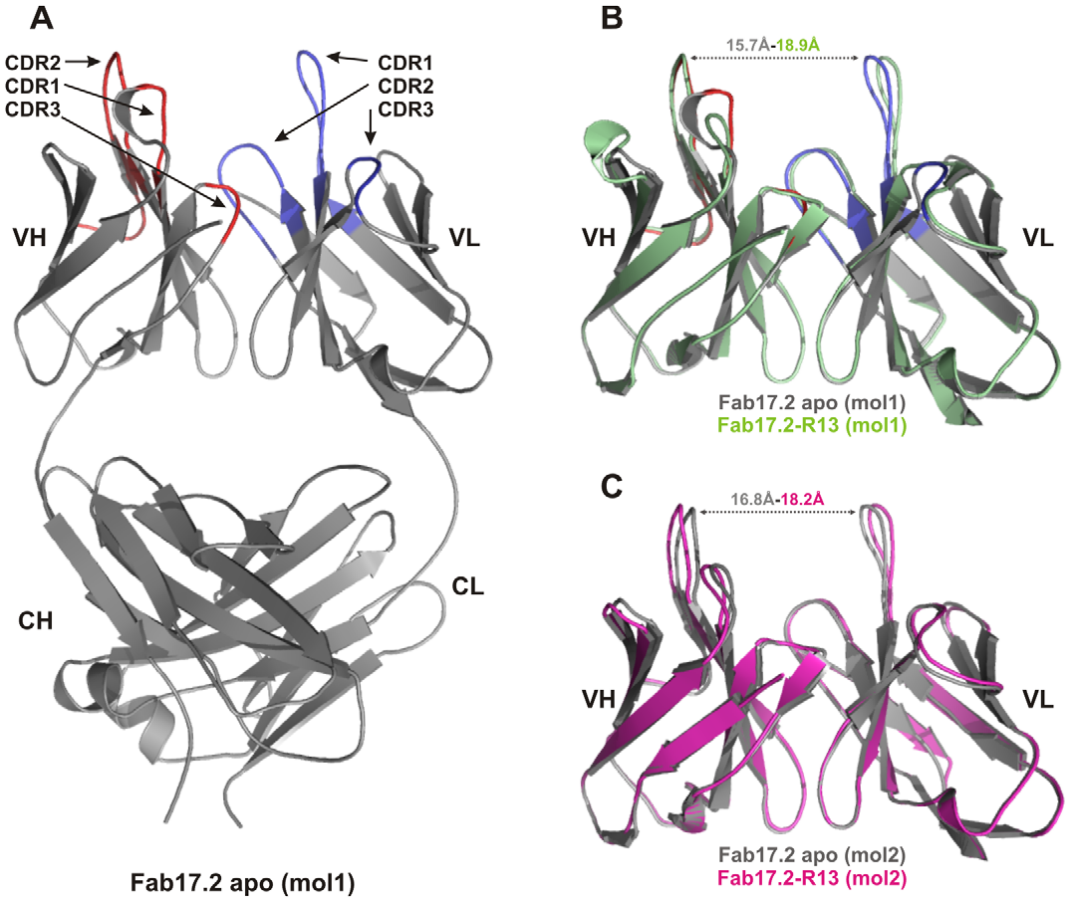
Structure of the Fab 17.2. A. Apo Fab 17.2 structure (molecule 1). Heavy chain CDRs are coloured in red and light chain CDRs in blue. B. Superposition of molecules 1, VH-VL region of the two crystals asymmetric units. C. Superposition of molecules 2, VH-VL region of the two crystals asymmetric units. Apo Fab 17.2 (grey), Fab 17.2-R13 molecule 1 (green) and molecule 2 (magenta).

**Figure 2:**
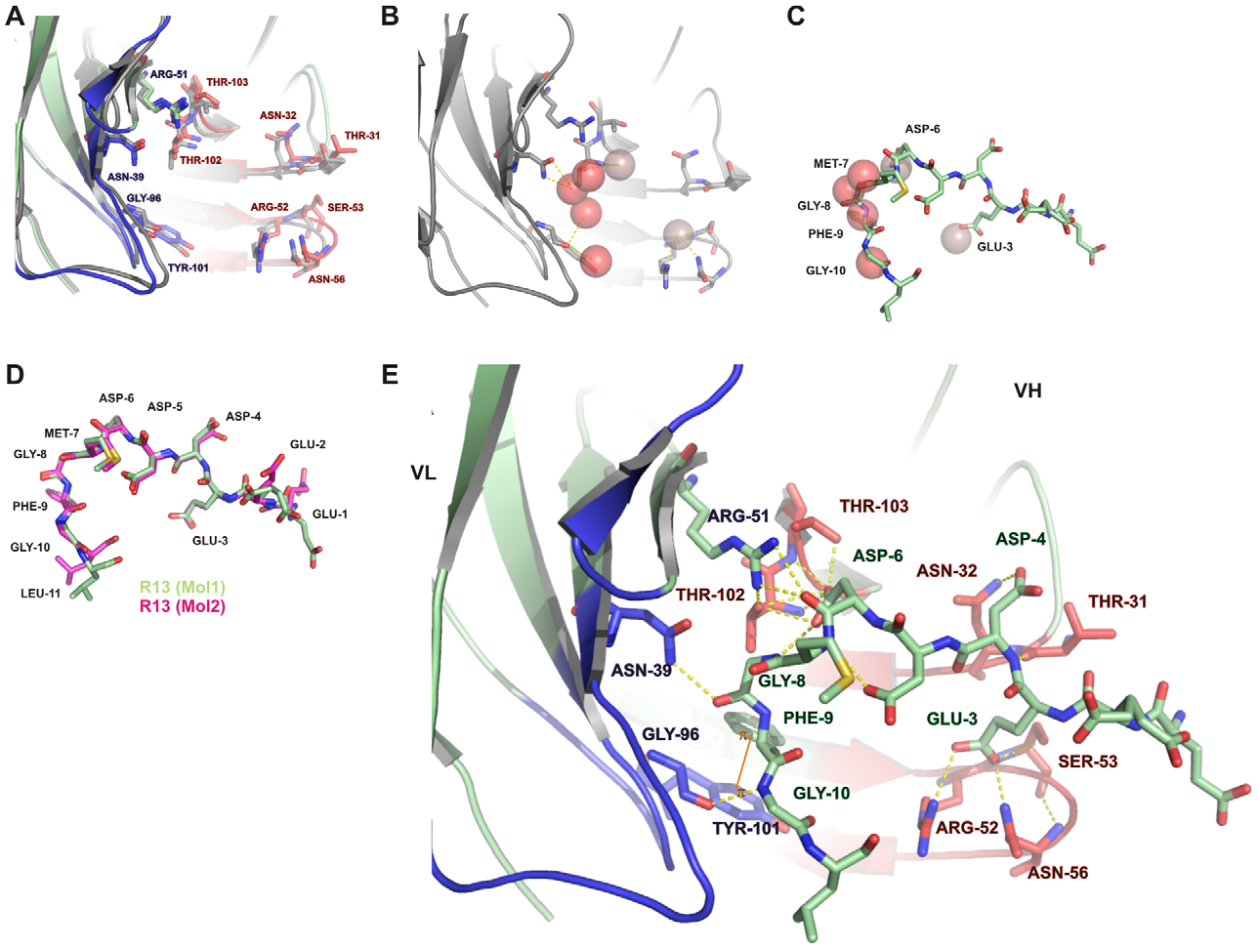
Structure of the Fab17.2 – R13 complex. A. Superposition of the apo Fab 17.2 and R13 complex structures (grey and green respectively). VH and VL contact residues are indicated. B. Main water molecules present on the antigen binding site of Fab 17.2 apo. C. Superposition of water molecules present in the apo Fab 17.2 that are replaced by the peptide in the Fab 17.2 R13 complex. D. Superposition of R13 peptides from molecules 1 (green) and 2 (magenta). E. Structure of the Fab 17.2-R13 complex (molecule 1). All hydrogen bonds between mAb 17.2 and peptide R13 are illustrated as dotted yellow lines. The π-stacking interaction between VL Tyr101 and the epitope Phe9 is also indicated. Heavy chain CDRs are coloured in red and light chain CDRs in blue the peptide is coloured in light green.

No significant structural differences were observed between individual variable domains. A pair-wise superposition of all VL domains from the apo and R13 complex Fab molecules gives a mean r.m.s. difference in Cα position of 0.28 Å (range 0.23–0.34 Å). Similarly, the superimposed VH domains give a mean r.m.s. difference in Cα positions of 0.28 Å (range 0.23–0.33 Å). For both VH and VL, the largest differences occur between the R13 complex and apo forms, but these are small, showing that the polypeptide conformation was largely conserved upon binding the antigen. The most notable structural change to occur upon binding R13 is a small relative rotation between the VL and VH domains, leading to an opening out at the antigen-binding site when the antigen is bound, with a 4.2° rotation from the apo to R13 complex state for molecule 1 and 4.6° for molecule 2. The distance between the tips of CDR-L1 and CDR-H2 is correspondingly shifted from 15.7 Å and 16.8 Å in the apo structure to 18.9 Å and 18.2 Å in the R13 complex in molecules 1 and 2, respectively (**Figure 1**). Interestingly, side chain conformations at the antigen binding site are entirely conserved upon binding the antigen (**Figure 2A**). In addition, there are six water molecules within the Fab 17.2 antigen combining site in the apo structure that are displaced by the peptide in the Fab 17.2 R13 complex (**Figure 2B**). Two of the water molecules in the apo structure are replaced by the side chains of Glu3 and Asp6, supporting the pivotal role of these residues in the antibody-antigen interaction. The remaining four waters are displaced by the main chain of the peptide, residues Met7 to Gly10 (**Figure 2C**). The complex structure also reveals two water-mediated interactions between the peptide and the CDR-H1 (Thr31 and Asn32) engaging both side and main chain atoms of a single epitope residue, Asp4.

The two bound R13 peptides from the complexes in the asymmetric unit are similar in structure, superimposing with an r.m.s. difference in Cα positions of 0.78 Å (1.06 Å for all main chain atoms). The peptide structures diverge most at the N- and C-termini, where contacts with the antibody are sparse, but are very similar in the central region of the peptide between residues Asp3 and Gly10 (**Figure 2D**). Antibody-peptide interactions are similar in the two independent Fab 17.2-R13 complexes and include 13 hydrogen bonds, four with VL and nine with VH, as well as a salt bridge between VH Arg52 and R13 Glu3 (**Table 2**). In addition, an aromatic stacking interaction occurs between VL Tyr101 and R13 Phe9. These results highlight the importance of some key contact residues on the epitope, such as Glu3, Asp6 and Phe9 (**Figure 2E**).

**Table 2:** Polar contacts between Fab 17.2 and R13 peptide.

Fab	CHAIN	CDR	Atom [MC/SC]			Ag
Asn39	L	1	Nδ2 [SC]	…	O [MC]	Gly8
Arg51	L	2	Nη2 [SC]	…	O [MC]	Asp6
Arg51	L	2	Nη1 [SC]	…	O [MC]	Asp6
Gly96	L	3	O [MC]	…	N [MC]	Gly10
Thr31	H	1	O [MC]	…	N [MC]	Asp4
Asn32	H	1	Nδ2 [SC]	…	Oδ1 [SC]	Asp4
Arg52	H	2	Nη1 [SC]	…	Oε2 [SC]	Glu3
Ser53	H	2	N [MC]	…	Oε1 [SC]	Glu3
Ser53	H	2	Oγ [SC]	…	Oε1 [SC]	Glu3
Asn56	H	2	Nδ2 [SC]	…	Oε1 [SC]	Glu3
Thr102	H	3	N [MC]	…	Oδ2 [SC]	Asp6
Thr102	H	3	Oγ1 [MC]	…	Oδ1 [SC]	Asp6
Thr103	H	3	N [MC]	…	Oδ2 [SC]	Asp6
Thr103	H	3	Oγ1 [SC]	…	Oδ2 [SC]	Asp6

MC: Main Chain; SC: Side Chain.

### Functional activity of the mAb 17.2

**Figure 3:**
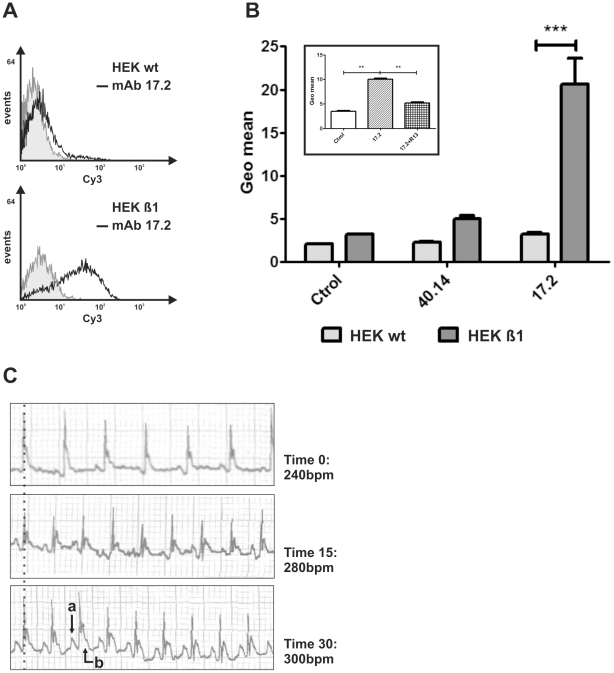
Functional activity of the mAb 17.2. A. Representative histogram showing the HEK and HEK-β1 cells labelled with mAb 17.2 followed by a Cy3-conjugated goat anti-mouse IgG. B. Results are expressed as means ± SD (n = 3). Inset: Binding of mAb 17.2 to HEK-β1 cells in the presence of R13 peptide. ** *p*<0.01; *** *p*<0.001. C. Passive transfer of mAb 17.2 to naïve mice. Repolarisation abnormalities (a) and first degree AV conduction block (b) are indicated by arrows. bpm: beats per minute.

The cross-reaction of anti-R13 Abs with β1-AR has been extensively reported [22], [23], [24], [34]. Indeed, the mAb 17.2, first described by Mahler et al (2001) [22], induced a dose-dependent increase on the beating frequency on neonatal rat cardiomyocytes culture that was abolished by bisoprolol, a specific β1-AR antagonist. Here, we show by flow cytometry that mAb 17.2 specifically recognized human β1-AR presented in a stable HEK-β1 cell line (**Figure 3A and B**). This cell line possesses a high receptor expression (800–1300 fmol/mg of total protein), as has been previously reported [35]. The mAb 40.14, which recognizes an internal epitope on TcP2β, gave no signal, indicating the specificity of mAb 17.2-β1-AR interaction (**Figure 3B**). In addition, this interaction was inhibited by pre-incubation of the mAb 17.2 with R13 peptide (**Figure 3B** **inset**). Furthermore, passive transfer of mAb 17.2 to naïve mice induced an increase in the beating rate from 240 bpm to 300 bpm after 30 minutes (**Figure 3C**). Repolarisation abnormalities (a) and first degree AV conduction block (b) were recorded at 15 and 30 minutes post-injection.

### Modelling the interaction with the human β1-AR

**Figure 4:**
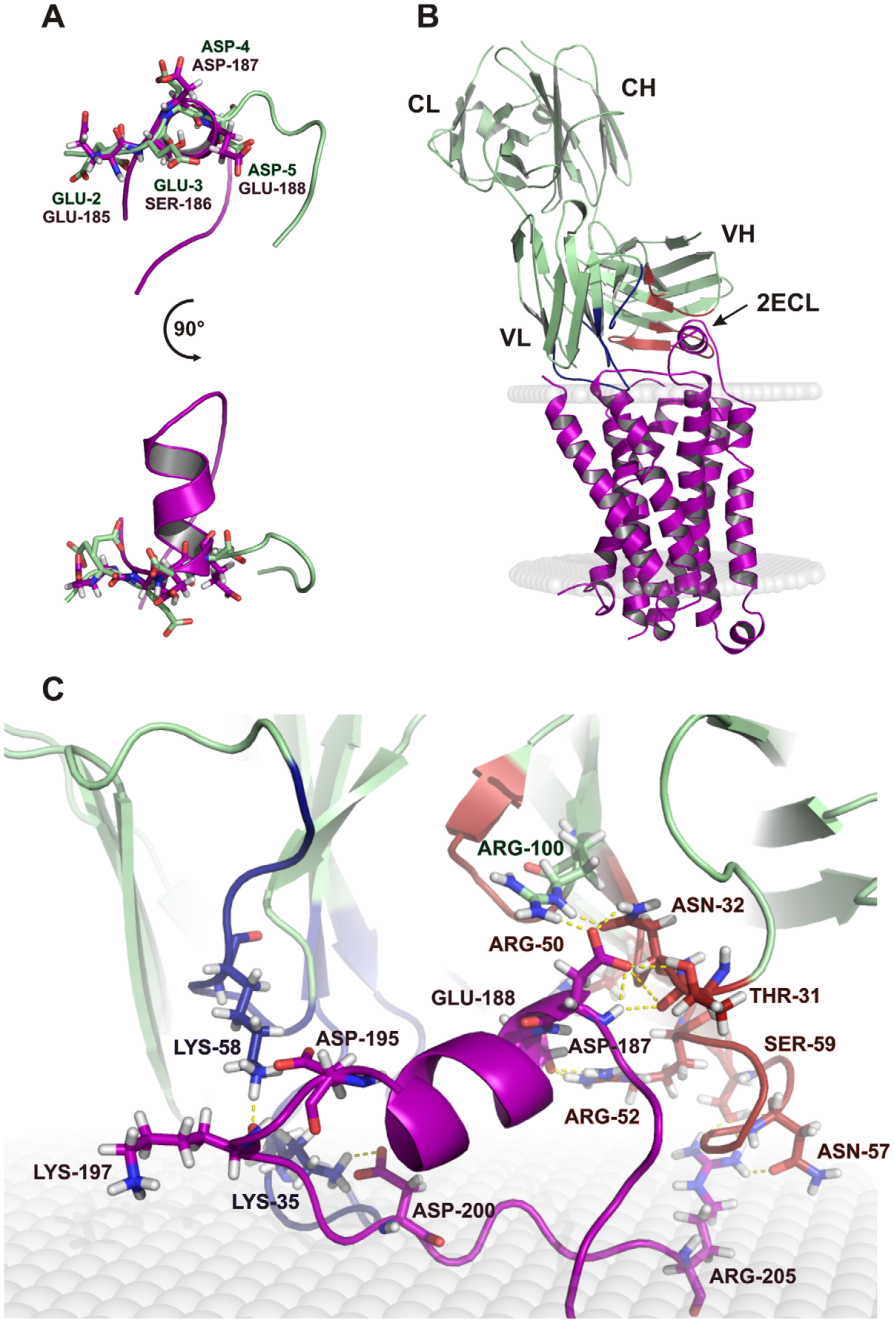
Model of the interaction of Fab 17.2 with the human β1-AR. A. Superposition of the second extracellular loop (2ECL) of the human β1-AR model (violet) and the region 2–5 of the epitope (light green). B. Complex of the Fab 17.2 (light green) with the human β1-AR (violet) inserted in a membrane model (grey). C. Zoom of the paratope region of 17.2 interacting with the second extracellular loop. The main contact points are illustrated as dotted yellow lines. Heavy chain CDRs are coloured in red and light chain CDRs in blue.

In view of the functional activity of the Ab on β1-AR, we generated a model of the interaction of the Fab 17.2 with the human receptor from the crystal structure of the turkey homologue. Briefly, we generated a model of the human β1-AR as described under material and methods. The structure of TcP2β epitope, R13, was aligned to the humanized second extracellular loop (2ECL) of the model to allow positioning the Ab on the putative binding site (**Figure 4A**) and the complex was subjected to a two minimization steps followed by heating, equilibration and production protocols under a distance-dependent, dielectric-implicit solvent model (**Figure 4B**). The interaction energy of the complex Ab-β1-AR was estimated at −430 kcal/mol. Polar contacts found in the model are summarized in **Table 3**. Some of Ab residues identified in the interaction with the humanized 2ECL peptide were key residues in the interaction with the *T. cruzi* epitope, such as Thr31, Arg52 and Asn56 of the heavy chain. In addition, the acidic residues present on the humanized β1-AR model (Asp187, Glu188, Asp195 and Asp200) established many hydrogen bonds with different residues of the Fab (**Figure 4C**). Remarkably, while the light chain established just a few contact points with the *T. cruzi* epitope, it established many hydrogen bonds with β1-AR, as can be seen in **Figure 4** **and** **Table 3**, suggesting an important role in stabilizing the interaction with the receptor.

**Table 3:** Polar contacts in the model between 17.2 and β1-AR.

mAb	CHAIN	CDR	Atom [MC/SC]			β-1AR
Gln27	L	1	Oε1 [SC]	…	Hη22[SC]	Arg323
Ser28	L	1	Oγ[SC]	…	Hη11[SC]	Arg323
Asp31	L	1	Oδ2 [SC]	…	Hω[MC]	Arg323
Ser32	L	1	Oγ[SC]	…	Hω[MC]	Val326
Lys35	L	1	Hζ1 [SC]	…	Oδ1 [SC]	Asp200
Lys35	L	1	Hζ1 [SC]	…	Oδ2 [SC]	Asp200
Lys35	L	1	Hζ2 [SC]	…	Oδ2 [SC]	Asp200
Lys58	L	2	Hζ1 [SC]	…	O [MC]	Lys197
Lys58	L	2	Hζ2 [SC]	…	Oδ1 [SC]	Asp195
Lys58	L	2	Hζ3 [SC]	…	Oδ1 [SC]	Asp195
Lys58	L	2	Hζ3 [SC]	…	Oδ2 [SC]	Asp195
His98	L	3	Hδ1 [SC]	…	O [MC]	Asp322
Thr31	H	1	Hγ1 [SC]	…	Oδ2 [SC]	Asp187
Asn32	H	1	Hδ22[SC]	…	Oδ1 [SC]	Asp187
Arg50	H	2	Hη22 [SC]	…	Oε1 [SC]	Glu188
Arg52	H	2	Hη12 [SC]	…	Oε2 [SC]	Glu188
Arg52	H	2	Hη22 [SC]	…	Oε2 [SC]	Glu188
Asn56	H	2	Oδ1 [SC]	…	Hη11 [SC]	Arg317
Asn57	H	2	Oδ1 [SC]	…	Hη11 [SC]	Arg205
Asn57	H	2	O [MC]	…	Hη12 [SC]	Arg205
Ser59	H	2	Oγ[SC]	…	Hη12 [SC]	Arg205
Ser59	H	2	Oγ[SC]	…	Hη22 [SC]	Arg205
Tyr61	H	2	Hη [SC]	…	O [MC]	Phe315
Arg100	H	3	Hη21 [SC]	…	Oδ1 [SC]	Asp187

MC: Main Chain; SC: Side Chain.

## Discussion

Alanine-mutation scanning analysis on the R13 epitope showed the importance of Glu3, Asp5, Asp6, Gly8 and Phe9 for mAb 17.2 recognition [22]. Crystal structure confirmed the interaction of Glu3, Asp6, Gly8 and Phe9 with the antibody and exposed a polar contact between Asp5 and Met7. This interaction may contribute to stabilize the conformation of the peptide that was shown to behave as random coil in solution [36].

Despite the sequence similarity between the epitope sequence (EEEDDDMGFGL) of TcP2β and the acidic region on the human second extracellular loop (AESDEA) of β1-AR, crystallographic data and modelling showed that this minimal region is involved but not sufficient for an effective interaction. The interaction energy between the Fab and the whole receptor was estimated at −430 kcal/mol. However, the interaction energy of the Ab with only the 2ECL was −283 kcal/mol, suggesting that other regions on the receptor contribute to stabilize the interaction within the complex. These data could also explain the low affinity of mAb 17.2, or single chain derived recombinant antibodies, with the β1-AR 2ECL peptide H26R measured by surface plasmon resonance [22], [25]. It is possible that electrostatic forces guide the first contacts between an electropositive antigen-binding pocket and the electronegative region of the 2ECL, with the heavy and light chains establishing additional contacts with the receptor. Comparison of the buried surfaces in both crystallized complex and the model (**Table 4**) shows a larger surface for the complex with the β1-AR, supporting this hypothesis. However, the shape complementarities for the model-Fab complex were smaller in comparison to R13 complex (**Table 4**), suggesting a reduced affinity for the β1-AR. Together, these data support the idea of a bystander activity of mAb 17.2 on the cardiac receptor rather than classical epitope mimicry of acidic residues.

**Table 4:** Buried surface upon complex formation, with shape complementarities (Sc) [50] and number of contacts observed between the mAb 17.2 and the R13 peptide in the crystal structure^1^ and the model^2^.

		Fab17.2 – R13^1^	Fab17.2 - β1AR^2^
Surface (Å^2^)	Ab (V_L_/V_H_)	648 (252.7/395.3)	1414.9 (868.3/546.6)
	Ag	845	2972.1
	Total	1493	4387
Sc		0.77	0.614
N° contacts	Polar	14	46
	Total	32	96

Recently, the crystal structure of turkey β1-AR in complex with different agonists and antagonists was published [37]. In this work, the authors showed the presence of structural differences between the 2ECL of the β1-AR bound to the agonist dobutamine (PDB 2Y01) and to the antagonist cyanopindolol (PDB2VT4). A 1 Å contraction of the ligand-binding pocket between helices H5 and H7 was observed in the agonist complex relative to the antagonist complex. The contraction of the catecholamine-binding pocket induced a conformational change in the 2ECL [37]. In addition, we previously reported the construction of two single chain recombinant antibodies (scFv) derived from the mAb 17.2, named scFv C5 and B7 [25]. Both scFv were able to recognize *T. cruzi* ribosomal P proteins and β1-AR in Western blot, ELISA, surface plasmon resonance and immunofluorescence. In functional assays, however, the monomeric scFv B7 behaved as a β1-AR antagonist, while the dimeric scFv C5 acted as a β1-AR agonist. The interaction of the Fab fragment with the β1-AR should thus correspond to an antagonist conformation. A dimeric interaction of the Ab with the receptor would, however, be constrained by the distance of the two antigen-binding sites on the dimeric Ab. Since β1-AR forms a family of dimeric membrane proteins, we suggested that the interaction constraints between the two dimers require a small rearrangement of the receptor dimer, decreasing the pharmacophore pocket, as has been described for partial agonists [37]. This could explain the experimental observation that monomeric fragments of Abs against the second extracellular loops of β1-AR act as inverse agonists while the bivalent fragments or dimeric antibodies behave as partial agonists [25], [38], [39], [40].

The clinical relevance of auto-reactive Abs in the context of the pathogenesis of cChHD is still controversial. Chronic Chagas' patients develop Abs against G protein-coupled receptors such as β1-AR, β2-AR and M2-AChR and there are different profiles of these Abs accordingly with the clinical outcome [23], [41], [42]. In the case of cChHD, there is a prevalence of β1-AR and M2-AChR Abs [43]. Interestingly these Abs can be detected before the clinical manifestations, supporting the hypothesis of Abs as pathogenic driver for clinical manifestations such as cardiomyopathy or megacolon [43]. It is well documented that chronic adrenergic stimulation mediated by antibodies may have a cardio-toxic effect resembling that caused by catecholamine, which induce cardiac changes similar to those observed in Chagas' disease; namely induction of micro focal lesions associated with a mononuclear cell infiltrate with increased involvement of the left ventricle, and particularly the left ventricular apex [44], [45], [46], [47], [48]. Moreover, IgGs from cChHD patients as well as the monoclonal antibody 17.2 were able to provoke apoptosis on HL-1 cardiac cell line, effect that was diminished by either R13 peptide or propranolol [26], reinforcing the pathogenic role of anti-R13 Abs acting on the β1-AR.

Our data provide a molecular basis for the understanding of the ß1-AR bystander activation by anti-R13 Abs. Furthermore, as severe cases of chronic Chagas' Heart Disease are related to high levels of Abs directed against the R13 epitope [8], [17], [18], [19], [20], [23], [49], and immuno-purified anti-R13 Abs from some of these patients showed in common that R13 residues Glu3, Asp6 and Phe9 were essential for recognition [23] (as was shown for mAb 17.2), we suggest that high blood concentrations of these antibodies may exert a systemic effect by inducing functional changes in cell types and tissues expressing this receptor, thereby increasing liability to chronic pathology from *T. cruzi* infection.
